# Can previously sedentary females use the feeling scale to regulate exercise intensity in a gym environment? an observational study

**DOI:** 10.1186/s13102-015-0023-8

**Published:** 2015-11-26

**Authors:** Charlotte C. Hamlyn-Williams, Gavin Tempest, Sarah Coombs, Gaynor Parfitt

**Affiliations:** Institute of Child Health, University College London, Population, Policy and Practice, UCL Institute of Child Health, 30 Guilford Street, London, WC1N 1EH UK; Sport and Health Sciences, College of Life and Environmental Sciences, St Lukes Campus, Heavitree Road, Exeter, EX1 2LU UK; Alliance for Research in Exercise, Nutrition and Activity, Sansom Institute, University of South Australia, 101 Currie St, Adelaide, SA 5001 Australia

**Keywords:** Affect, Physical activity, Exercise prescription, Ventilatory threshold, Ideographic responses

## Abstract

**Background:**

Recent research suggests that the Feeling Scale (FS) can be used as a method of exercise intensity regulation to maintain a positive affective response during exercise. However, research to date has been carried out in laboratories and is not representative of natural exercise environments. The purpose of this study was to evaluate whether sedentary women can self-regulate their exercise intensity using the FS to experience positive affective responses in a gym environment using their own choice of exercise mode; cycling or treadmill.

**Methods:**

Fourteen females (24.9 years ± 5.2; height 166.7 ± 5.7 cm; mass 66.3 ± 13.4 kg; BMI 24.1 ± 5.5)) completed a submaximal exercise test and each individual’s ventilatory threshold ($\dot{V}T$) was identified. Following this, three 20 min gym-based exercise trials, either on a bike or treadmill were performed at an intensity that was self-selected and perceived to correspond to the FS value of +3 (good). Oxygen uptake, heart rate (HR) and ratings of perceived exertion (RPE) were measured during exercise at the participants chosen intensity.

**Results:**

Results indicated that on average participants worked close to their $\dot{V}T$ and increased their exercise intensity during the 20-min session. Participants worked physiologically harder during cycling exercise. Consistency of oxygen uptake, HR and RPE across the exercise trials was high.

**Conclusion:**

The data indicate that previously sedentary women can use the FS in an ecological setting to regulate their exercise intensity and that regulating intensity to feel ‘good’ should lead to individuals exercising at an intensity that would result in cardiovascular gains if maintained.

## Background

Research indicates that positive affective responses experienced during exercise may enhance future exercise behaviour [1, 2]. However, identifying an optimal exercise intensity that elicits a positive affective response in all individuals is challenging. One method to overcome this problem is to allow individuals to self-regulate their exercise to an intensity that they prefer [3–5]. Research using this method has shown that, on average, individuals choose to exercise at intensities around their ventilatory threshold ($\dot{V}T$; the transition from aerobic to anaerobic metabolism) [6] or lactate threshold (LT), and that while intensity remains within this range, evidence has shown that typically, participants start below these metabolic landmarks and then choose to increase their workrate/intensity. The reasons suggested for this include that it is the result of a natural warm-up strategy [7], or that it takes time to locate an intensity that is ‘preferred’ [3], or that over time participants adjust to the metabolic requirements and as they become comfortable choose to increase the intensity [5]. However, the corresponding affective responses have remained stable [3, 4, 7]. Further, this method of allowing participants to self-regulate their intensity has resulted in reduced inter-individual variability in affective responses, making self-regulation of exercise based on preference a more appropriate method of exercise prescription for eliciting positive affective responses than the traditional ‘relative’ intensity protocols. Rose and Parfitt [8] suggested that one approach that would remove this variability altogether would be to allow the individual to self-regulate their exercise intensity to result in a specific positive affective response (affect-regulate). To date, three studies have sought to explore the feasibility of using affective anchors to guide the self-regulation of exercise using the Feeling Scale (FS) [8–10]. Rose and Parfitt [8] asked seventeen women to complete eight 30-min exercise sessions at an intensity they perceived to correspond to FS + 1 (fairly good) and FS + 3 (good). On average participants worked 2–4 % above $\dot{V}T$ in the first exercise session and between 6–10 % above $\dot{V}T$ in the three subsequent sessions. Irrespective of condition (FS + 1 or FS + 3) individuals chose to increase their exercise intensity across the 30 min. This pattern was reflected in the heart rate (HR) and rating of perceived exertion (RPE) and the selected intensity was consistent both within and across trials. A limitation of this study, however, was that $\dot{V}T$ was inferred from a prior graded exercise test (GXT) during which oxygen uptake and HR were recorded.

Parfitt et al. [9] sought to overcome these limitations and objectively measured the physiological cost of the exercise intensity associated with FS + 1 (fairly good) and FS + 3 (good) in active females. When referenced relative to $\dot{V}T$ the exercise intensity chosen by individuals was close to $\dot{V}T$, and as with the previous study, increased over the 20-min exercise period to maintain the target affective response. Whilst there are differences between these two studies, results indicate that participants are exercising at an intensity that should improve fitness and health benefits if maintained. This has now been confirmed in a study that required sedentary women to affect-regulate their exercise intensity during supervised training twice a week for 8 weeks [10].

The primary aim of this study was to examine if affect-regulated exercise could be utilised by previously sedentary individuals in a natural environment: a fitness gym. Additionally, the study aimed to overcome previous study limitations. This study allowed participants to choose their exercise mode, as choice, or rather the lack of choice, has been shown to impact affective responses [11–14]. Additionally, in line with Buscombe and Inskip, participants were permitted to alter the intensity at any stage during the course of the workout [14]. Finally, based upon Parfitt et al. [9] participants completed three exercise trials at the same target affective response (FS + 3 good) to examine consistency across the three sessions. It was hypothesised that 1) individuals would self-select an intensity of exercise on their chosen mode that lay within the range recommended by the ACSM for health improvements [8, 15, 16]; 2) exercise intensity would increase across the exercise sessions [3, 5, 8, 9]; and 3) participants would be able to self-regulate with high consistency across sessions. [8, 9]

## Methods

### Participants

Fourteen female volunteers (mean ± SD; age 24.9 ± 5.2 years; height 166.7 ± 5.7 cm; mass 66.3 ± 13.4 kg; BMI 24.1 ± 5.5) participated in the study. Participants were recruited through poster advertisement on a University campus and convenience sampling. To be eligible, participants had to be female and sedentary (no participation in exercise more than once a week for the previous six months). All participants read and signed informed consent forms approved by the University Ethics Committee prior to participation. Participants completed a Physical Activity Readiness Questionnaire (PAR-Q) and were healthy, non-smokers, and free from muscular-skeletal injury.

### Measurement instruments

Affective valence (pleasure/displeasure) was assessed using the FS [17]. Participants were asked to exercise so that they felt ‘good’ on the 11-point bipolar scale, which ranges from +5–5, with verbal anchors of very good (+5), good (+3), fairly good (+1), neutral (0), fairly bad (−1), bad (−3), and very bad (−5). Participants were given standardised instructions on how to use the scale and had time to practice during the familiarisation stage and the submaximal exercise test. The FS has previously been shown to be a valid measure of affect [18, 19].

The Borg 6–20 RPE scale [20] was used to assess whole-body exertion. Participants stated the number that reflected how hard the exercise felt on a 6–20 scale, ranging from no exertion at all (6) through somewhat hard (13) to maximal exertion (20). Participants were given standardised instructions on how to use the scale and had time to practice during the familiarisation stage.

A portable gas analyser (Cosmed K4, Rome, Italy) complete with a face mask was used to measure expired gases throughout the duration of the testing. The K4 has been shown to provide valid measurements of oxygen uptake across a range of exercise intensities [21]. The K4 was calibrated before every test in accordance with manufacturer’s guidelines.

A Polar HR monitor (Polar Electro, Finland) was used to measure HR throughout the exercise sessions. Heart rate was recorded continuously using a wireless chest strap telemetry system with a watch worn on the right wrist. Heart rate was blinded from the participant during the exercise sessions.

### Procedures

Height and mass measures were taken upon arrival at the laboratory. Participants were briefed on procedures and any outstanding questions were addressed. Maximal HR (HRmax) was calculated using Tanaka’s equation (208–0.7 * age) [22]. Participants were given the choice of using either the treadmill (Integrity Series, Life Fitness, UK) or cycle ergometer (Lifecycle 9100, Life Fitness, UK) and were offered a brief trial on each piece of equipment before making a decision. Participants were given standardised instructions on how to use the scales in the forthcoming submaximal exercise test. A period of familiarisation and practice with the scales was provided during the warm-up period.

### Submaximal exercise test

All participants performed a submaximal exercise test on their chosen mode of exercise. A submaximal exercise test (up to 85 % HRmax) was deemed most appropriate given that all participants were sedentary and may have been unfamiliar with the feelings and sensations associated with maximal exercise. Additionally, the cessation of the exercise test at 85 % of HRmax would reduce the potential negative affect associated with the higher exercise intensities [23–25] which may impact participant recruitment. Participants FS responses and RPE were recorded every minute during exercise.

#### Cycling exercise

Participants completed a three-minute warm-up (15 W) followed by a step protocol starting at 25 W and increasing by 10 W^−1^ (pedal cadence was maintained at 50 RPM). The end point of the exercise test was marked by the participant reaching 85 % of their age-related predicted HRmax.

#### Treadmill exercise

Participants completed a three-minute warm up, walking or running at a comfortable pace, followed by the Balke-Ware treadmill (step) protocol. A speed of 5.4 km/h was maintained and a starting gradient of 2.0 % was increased by 1 % every minute. The termination criteria were the same as for the cycling exercise.

### Determination of maximal oxygen uptake ($\dot{V}{0}_2 \max$) and Ventilatory Threshold ($\dot{V}T$)

Using data recorded at one minute intervals throughout the submaximal exercise test, maximal oxygen uptake ($\dot{V}{0}_2 \max$) was determined by plotting HR against oxygen uptake ($\dot{V}{O}_2$ ml^.^min^-1.^kg^−1^). Linear regression analysis was then used to extrapolate to each individual’s HRmax. The $\dot{V}T$ was determined using visual determination and agreement between the three-method (ventilatory equivalent; excess carbon dioxide; and modified V slope) procedure proposed by Gaskill et al. [26].

### Gym-based exercise sessions

Participants completed three 20-min exercise sessions in a fitness gym. Guidelines indicate 10-min of continuous aerobic should be performed for health gains [27], with a total of 30-min a day recommended. However, as the participants in the current study were sedentary 20-min sessions were chosen so as not to deter the participants from adhering to the study. The ACSM recommend that, dependent on the individuals’ fitness, exercise sessions should be of moderate duration, progressing up to 30-min as adaptation to training occurs [16]. Each session was held at least 48 h apart, and at the same time of day when possible. The mode of exercise in the gym sessions corresponded to that of the submaximal exercise test. Participants were instructed to aim to work at an intensity which reflected FS + 3 (good). The portable gas analyser was fitted to the participant to continuously measure metabolic data and a HR monitor. Participants completed a three-minute warm-up, to prepare for exercise and to enable them to find an initial intensity which reflected FS + 3. During the test participants were able to adjust the exercise intensity (altering the gradient or speed on the treadmill, or resistance on the exercise bike) at any stage, although the display and values themselves were kept blind. At five-minute intervals, HR was recorded and participants were asked to confirm that they were exercising at FS + 3 and indicate their RPE using visual scales placed in front of them.

Upon completion of all exercise sessions open-ended qualitative questions were asked to explore participant’s experiences of participating in the study and using the Feeling Scale to regulate their exercise intensity.

### Data analysis

The $\dot{V}{0}_2$ and HR data were converted to a proportion of the value recorded at $\dot{V}T$. The representation of data relative to each individual’s $\dot{V}T$ is abbreviated as VO2 expressed as a percentage of VO2 at VT ($\dot{V}{0}_2$ as % $\dot{V}{0}_{2VT}$) and HR as a % of that achieved at VT (%HR at $\dot{V}T$). Using SPSS 15.0 for windows (SPSS Inc., Chicago, IL), a series of three factor, trial (1, 2, 3) by mode (cycling, treadmill exercise) by time (5–10–, 15–20-min) analyses of variance (ANOVA) with repeated measures (trial and time) were conducted for $\dot{V}{0}_2$ as % $\dot{V}$ O_2VT_ and %HR at $\dot{V}T$ and RPE. Greenhouse Geisser corrections were applied if the assumption of sphericity was not met. All significant main and interaction effects (*p* < .05) were followed by Bonferroni adjusted pairwise comparisons. Intraclass correlation coefficients (ICC) were calculated for $\dot{V}{0}_2$ as % $\dot{V}{0}_{2VT}$ and %HR at $\dot{V}T$ and RPE to examine the consistency in the intensity chosen across trials at each time point (5-, 10-, 15- and 20-min).

## Results

Following the GXT, to ensure there were no differences in physiological profiles ($\dot{V}{0}_2$ as % $\dot{V}{0}_{2VT}$ and %HR at $\dot{V}T$, $\dot{V}{0}_2$% $\dot{V}{0}_2 \max$, and predicted $\dot{V}{0}_2 \max$) between participants in cycling and treadmill exercise groups, independent t-tests were conducted. No differences in $\dot{V}{0}_2$ as % $\dot{V}{0}_{2VT}$, %HR at $\dot{V}T$, $\dot{V}{0}_2$% $\dot{V}{0}_2 \max$ or predicted $\dot{V}{0}_2 \max$ were recorded.

### Oxygen uptake

A significant main effect of mode (F(1,12) = 5.28, *p* < .05) was recorded for $\dot{V}{0}_2$ as % $\dot{V}{0}_{2VT}$. $\dot{V}{0}_2$ as % $\dot{V}{0}_{2VT}$ was higher during cycling (120.2 ± 23.1; above $\dot{V}T$) than treadmill exercise (89.1 ± 27.3; below $\dot{V}T$), but remained stable across all time points; 5-, 10-, 15- and 20-min (*p* > .05; see Table 1).

**Table 1:** Means ± standard deviations of $\dot{V}{0}_2$ as % $\dot{V}{0}_{2VT}$, %HR at $\dot{V}T$ and RPE at each time point by mode

	Mode	Time			
		5	10	15	20
$\dot{V}{0}_2$ as % $\dot{V}{0}_{2VT}$	Cycling	116.4 ± 20.9	120.7 ± 20.7	124.2 ± 22.3	119.3 ± 31.7
	Treadmill	85.7 ± 25.8	88.5 ± 27.4	90.8 ± 28.2	91.3 ± 28.7
	Overall	101.1 ± 27.6	104.6 ± 28.7	107.5 ± 30.0	105 ± 32.5
%HR at $\dot{V}T$	Cycling	106.9 ± 14.4	114.3 ± 12.5	117.4 ± 12.3	118.4 ± 12.2
	Treadmill	98.7 ± 15.7	103.9 ± 18.6	108.0 ± 23.1	106.4 ± 18.4
	Overall	102.8 ± 15.1	109.1 ± 16.1	112.7 ± 18.4	112.4 ± 16.2
RPE	Cycling	11.5 ± 0.9	12.2 ± 0.7	12.7 ± 0.8	12.6 ± 1.3
	Treadmill	10.3 ± 108	11.4 ± 1.7	12.0 ± 1.6	12.0 ± 1.4
	Overall	10.9 ± 1.5	11.8 ± 1.4	12.3 ± 1.3	12.3 ± 1.4

### Heart rate

A significant main effect of time (F (2,21) =18.6 *p* < .001) was recorded for %HR at $\dot{V}T$. Percentage HR at $\dot{V}T$ significantly increased from 5–10 min, but remained stable at 10–15- and 20- min (see Table 1).

### Rating of perceived exertion

A significant main effect of time (F (3,36) = 23.0, *p* < .001) was recorded for RPE. Perceived exertion significantly increased from 5–10- to 15-min, but then remained stable until 20-min (see Table 1).

Consistency across and within trials for $\dot{V}{0}_2$ as % $\dot{V}{0}_{2VT}$ and %HR at VT was high during both cycling (range .89–95 and .81–92, respectively) and treadmill exercise (range .87–93 and .91–99, respectively; see Table 2. Consistency across and within trials for RPE was not quite as high (although still relatively good) during cycling (range .58–84 and .73–98, respectively) and treadmill exercise (range .76–95 and .69–98, respectively).

**Table 2:** Intraclass correlations of $\dot{V}{0}_2$ as % $\dot{V}{0}_{2VT}$, %HR at $\dot{V}T$ and RPE across trials 1, 2, and 3 at each time point by mode

	Mode	Time			
		5	10	15	20
$\dot{V}{0}_2$ as % $\dot{V}$ O_2VT_	Cycling	.89	.95	.90	.94
	Treadmill	.87	.93	.93	.92
%HR at $\dot{V}T$	Cycling	.92	.88	.83	.81
	Treadmill	.94	.96	.92	.91
RPE	Cycling	.84	.58	.81	.74
	Treadmill	.95	.76	.89	.89

## Discussion

The purpose of this study was to provide preliminary evidence for the practical use of the FS to regulate exercise intensity in a natural gym environment, as previous research has been restricted to a laboratory setting. Sedentary females were generally able to self-regulate their exercise intensity using the FS during 20-min cycling or treadmill exercise in a gym environment, although data suggest that there is a mode effect and that there may be a need to balance physiological and psychological considerations.

In support of the first hypothesis, individuals self-selected an exercise intensity using their chosen mode that lay within the range recommended by the ACSM for health improvements. The exercise intensity equated to 72 % predicted HRmax (cycling 75 % and treadmill exercise 69 % predicted HRmax) that lies within the ACSM [28] recommended range, which extends from a low of 64–70 % to a high of 90 % HRmax. Heart rate data from the current study were similar to previous laboratory investigations with self-regulated exercise based on affective feelings [8, 9]. Rose and Parfitt [8] found that sedentary women selected an intensity equating to 70 % HRmax at FS + 3; only slightly lower than that of the current study.

The $\dot{V}{0}_2$ data paint a slightly different picture with an average exercise intensity of 58 % $\dot{V}{\mathrm{O}}_2 \max$ (cycling 66 % and treadmill exercise 50 % $\dot{V}{0}_2 \max$). Whilst within the ACSMs [28] recommended range of between 50–85 %, the wide inter-individual differences, which ranged between 42–79 % of $\dot{V}{0}_2$ max suggest that some participants (primarily during treadmill exercise) were not quite working at the recommended level of intensity necessary for the accrual of health benefits. However, $\dot{V}{0}_2$ max was only predicted for the participants in this study and therefore may not be as accurate as within previous studies. Thus, instead of focusing on % $\dot{V}{0_2}_{\max }$, potentially of more relevance to this study and future research is the % above or below $\dot{V}T$ that the individual is working. Research has indicated that when exercise prescription is based on the individually determined $\dot{V}T$, exercise is more effective in improving indices of fitness or health compared to traditional prescriptions [29, 30]. Results from this study revealed that only the participants cycling worked consistently above their $\dot{V}T$; an intensity shown to have a beneficial effect on health. Individual data show that all the participants who cycled achieved their $\dot{V}T$ for at least 5 min (the majority for 15 min) during the session. This was not the case for the participants who chose to exercise on the treadmill, with only 3 of the 7 exercising above or close to their $\dot{V}T$. These findings are contrary to the previous treadmill-based research [8, 9] and suggest that exercise mode influences the intensity of exercise selected by sedentary females in a gym environment. The explanation for this is not clear, although it may be that in the gym environment the females felt more self-conscious; particularly as participants reported that running wearing the K4 breath analyser was cumbersome and made it difficult to run properly. The K4 breath analyser is a ‘one-size fits all’ model, meaning that the same harness was worn by all participants despite differences in size and body shape. As a consequence, the analyser was more secure on some participants than others; something that became more pronounced when running compared to walking. These points were reflected in the post-study qualitative data:

*‘When I started running the machine on my front was really annoying. It kept getting in my way.’*

*‘People were staring at me.’*

This was not an issue on the bike as one’s torso remains relatively still during cycle. Alternatively it may be linked to individual differences in one’s preference and tolerance of high and low intensity exercise; a concept discussed by Ekkekakis et al. [31] who say that individuals differ in the intensity of exercise they prefer and the intensity they can tolerate. This could have a direct influence on the intensity an individual chooses when trying to maintain a state of feeling ‘good’. Future studies should seek to examine this concept further to identify if individual differences in tolerance and preference account for the intensities selected, as previous research has shown that preference can predict the intensity an individual will self-select [32–34].

The average RPE selected was 11.8 (cycling 12.2 and treadmill exercise 11.4), almost identical to that found by Rose and Parfitt [8] who reported an average of 11.4. These RPE scores are slightly lower than those recommended for the regulation of moderate intensity exercise (12–14) [27], however, in self-regulated exercise it is not uncommon for RPE ratings to become uncoupled from physiological variables [15, 35–37]. Previous research has shown perceptions of intensity are lower during self-regulated exercise compared to prescribed intensity exercise even when the actual intensity does not differ [38].

Numerous studies that have investigated affect-regulated exercise have reported that individuals spontaneously increase their intensity across time [3, 5, 7, 8]. However, in this study, the data are inconsistent and dependent on which physiological variable is considered. The $\dot{V}{0}_2$ remained stable with no time main effect recorded, but there was a significant increase for HR from 5–10 min and for RPE in the first 15 min. The lack of a time main effect for $\dot{V}{0}_2$ could have been due to the large variability in response in this variable, while the significant time main effects (for HR and RPE) would be explained as participants taking time to find the intensity to match feeling ‘good’ [32]. Qualitative data collected upon completion of all exercise sessions supported this explanation.

*“[I] gradually increased [the intensity] in order to reach an intensity that felt ‘good’. This ensured that I progressed slowly to a comfortable pace.”*

Finally, hypothesis three investigated the consistency of self-regulation across exercise sessions. Rose and Parfitt [8] argue that for the FS to be considered a reliable method of exercise regulation it is important to show high consistency across trials with regards to the relationship between certain intensities and particular affective states. Results from Rose and Parfitt [8] revealed that after one familiarisation session there was high consistency across exercise trials. Data from the current study suggests that individuals can regulate with high consistency from the first session as ICCs across trials and within trial, at each time point, were high.

As this study moved away from a laboratory setting into a gym environment it is important to note that the social context may have influenced the selection of exercise intensities. In some instances the gym was empty, but other times there were more people which could have encouraged modification of intensity selected. Similarly, environmental distractions, such as different music or TV may have influenced individual responses. Qualitative data from the current study revealed that promoting exercise in a real-world, ecologically valid setting, may be an important factor that influences the exercise-affect relationship. Participants found that the gym environment meant that they were less aware of what they were doing compared to if they had been exercising in a laboratory setting. One participant commented that she “*could just watch others exercising within the gym, and not worry about what [she] was doing”* with another stating that she was “*aware that [she] could just listen to the music, which helped with [her] pace*”. Watching other people exercising, and listening to music and movement surrounding the participants helped to distract them from physiological cues, and may have encouraged individuals to select higher exercise intensities, but may also have been a cause for variability as the environmental influences may have differed in each session. Therefore, using the FS in an ecological setting, such as the gym environment, may encourage individuals to select higher exercise intensities, or cause variability between sessions as they may be more distracted from the physiological cues that contribute to a reduction in exercise intensity [5] and affective responses [38, 39].

## Conclusion

These findings suggest that using the FS in an ecologically valid setting allows individuals to exercise at an intensity around their $\dot{V}T$, whilst feeling ‘good’, particularly on a cycle ergometer. The findings support and build on previous laboratory-based studies, and show that affective anchors can be used to self-regulate exercise sessions with the potential to then influence future exercise participation [1, 2]. Taking this approach for exercise prescription and promotion has the potential to ensure participants feel good during exercise and thus choose to continue to exercise, even if at a lower intensity than recommended. In comparison, other prescriptive approaches focusing on exercise intensity rather than the affective experience may initially have greater physiological gains, but may be experienced as unpleasant and thus discourage further exercise participation.

## Abbreviations

ACSM: American College of Sports Medicine
BMI: Body Mass Index
FS: Feeling Scale
GXT: Graded Exercise Test
HR: Heart Rate
ICC: Intraclass Correlations
LT: lactate threshold
RPE: Rating of Perceived Exertion
RPM: Revolutions per Minute
$\dot{V}{0}_2$: maximal oxygen uptake
$$ \dot{V}T $$: Ventilatory Threshold
W: watts
